# The consequences of job crafting and engagement in the relationship between passion for work and individual performance of Portuguese workers

**DOI:** 10.3389/fpsyg.2023.1180239

**Published:** 2023-08-18

**Authors:** Joana Vieira dos Santos, Alexandra Gomes, Diana Filipe Saraiva Rebelo, Luis Felipe Dias Lopes, Martiele Gonçalves Moreira, Deoclécio Junior Cardoso da Silva

**Affiliations:** ^1^Centro de Investigação em Psicologia (CIP, UAlg), Lisbon, Portugal; ^2^Faculdade de Ciências Humanas e Sociais, Universidade do Algarve, Faro, Portugal; ^3^Centro de Ciências Naturais e Exatas (CCSH, UFSM), Programa de Pós-graduação em Administração, Universidade Federal de Santa Maria, Santa Maria, Brazil

**Keywords:** harmonious and obsessive passion, job crafting, engagement, performance, work

## Abstract

**Introduction:**

This study sought to relate the two types of work passion, harmonious passion and obsessive passion, to the organizational consequences of engagement, job crafting, and perceived individual job performance. This study was based on the Employee Work Passion Appraisal model and conducted to evaluate possible statistical associations of the dualistic approach of passion used as an antecedent of positive and negative organizational outcomes (engagement, job crafting, and perceived individual job performance).

**Methods:**

The data collection and analysis for this study were accomplished by a transversal and quantitative study design. A non-probabilistic method was used to select a convenience sample composed of 305 Portuguese workers and was collected online from March to October 2020. The proposed hypotheses were evaluated using partial structural equation models.

**Results:**

Overall, the results supported the proposed hypotheses and showed that harmonious passion positively affected organizational outcomes, while obsessive passion negatively affected these outcomes; notably, our findings also revealed high individual performance, high obsessive passion, and consequently, a significant increase in structural labor resources, a significant decrease in harmful labor demands, and high absorption.

**Discussion:**

The findings highlight the importance of distinguishing between harmonious passion and obsessive passion in understanding their consequences for organizational outcomes. Promoting harmonious passion while managing the potential negative effects of obsessive passion is crucial for enhancing positive job-related behaviors and performance. Future research should explore interventions and strategies to foster harmonious passion, mitigate the negative impacts of obsessive passion, and ultimately improve overall work engagement and performance.

## 1. Introduction

Work is a crucial part of an individual’s life because it is where they spend most of their lives, both in terms of the workplace and in the extra hours devoted to this activity. For some, work is seen as a form of acquiring monetary income or reaching a higher position in the social hierarchy, while others value their work to the point of considering it a vocation (Wrzesniewski et al., 1997). For people whose work is important in their lives, it gives meaning to their existence (Wrzesniewski et al., 2003), becoming part of their identity (Vallerand and Houlfort, 2003). Thus, it is essential to study the concept of passion for work.

Passion for work can be defined as “an individual’s persistent, emotionally positive state, based on a sense of well-being, resulting from cognitive and affective evaluations arising from various professional and organizational situations that result in consistent and constructive work intentions and behaviors” (Zigarmi et al., 2009, p. 310).

The Employee Work Passion Appraisal model (Zigarmi et al., 2009), which focuses on a dualistic approach to passion, divides it into harmonious and obsessive passions (Vallerand et al., 2003). This approach will be used as the groundwork for our study since these two types of passion for work culminate in positive or negative work intentions. Engagement will be measured as an organizational consequence of the two types of passion for work since passion for work is a work attitude related to the physical, emotional, and cognitive aspects of work engagement (Kahn, 1990). Its presence in an organization broadens individuals’ perceptions and cognitions of their surroundings, giving workers the opportunity to identify the resources around them more effectively, and it also leads them to being better equipped and more strongly motivated to successfully undertake job crafting efforts (Halbesleben, 2011; De Beer et al., 2016).

Job crafting, for the majority of workers, leads to opportunities for improved workplace life and the possibility of meaningful contribution to the workplace (Grant and Parker, 2009). In this sense, it will also be measured as consequential as workers can create opportunities for passion for work through partial methods of job crafting (Berg et al., 2008 cit. in Teng, 2019). In this way, they are willing to invest time, attention, and energy in tasks of personal interest, take on extra tasks, or reshape their personal work goals (Berg et al., 2010). Finally, employee job performance denotes a crucial factor in determining an organization’s performance (Edwards, 1991). Therefore, it will also be an organizational consequence to be measured, because depending on the type of passion experienced by an employee, they may or may not experience performance benefits (Ho et al., 2011).

Thus, this research aims to investigate the explanatory contribution of the two types of passion for work, harmonious passion and obsessive passion, on the organizational consequences of engagement, job crafting, and perception of job performance. According to the literature, different types of passion culminate in different work intentions, whether more positive or negative; we seek to better understand their explanatory contribution to job crafting and job performance.

## 2. Theoretical background

The concept of work passion, as defined by Zigarmi et al. (2009), refers to an emotionally positive state of perseverance in individuals, rooted in a sense of well-being. It is a result of cognitive and affective evaluations stemming from different work and organizational contexts, leading to consistent and constructive work intentions and behaviors. The Employee Work Passion Appraisal model assumes cognitive and affective appraisals of various professional and organizational situations in the employee work environment that culminate in consistent and constructive work intentions (Zigarmi et al., 2009). With this, these evaluations refer to organizational characteristics such as procedural justice, distributive justice, growth, and performance expectations; on the other hand, work characteristics such as autonomy, task variety, workload balance, and meaningful work; and finally, relationship characteristics such as feedback, collaboration, and connection with colleagues and the leader (Zigarmi et al., 2009; Thibault-Landry et al., 2018). It is also noteworthy that these evaluations result from an ongoing process of evaluation. Individuals, by making continuous evaluations of work characteristics (work cognitions), consequently form conscious intentions that will be triggered based on meaning, i.e., mental representations of behavior that one can use to cope with the prior evaluation, allowing for more motivated and persistent behavior towards the organization and employees’ job roles and good long-term performance in organizational and work role behaviors (Zigarmi et al., 2009).

To contribute to passion for work, organizations must be able to provide meaning, independence, and flexibility for growth, recognition, and a sense of connectedness (Permarupan et al., 2013). With this knowledge, after much research, passion for work was categorized into eight elements: meaningful work, collaboration, fairness, autonomy, recognition, growth, connection with the leader, and connection with colleagues. If these elements are taken into consideration by the leader, it will consequently have an influence on employees’ passion for work in relation to their tasks and work performed (Blanchard, 2009, cited in Permarupan et al., 2013).

Passion is defined as a strong inclination towards an activity that the individual enjoys, which has meaning their life and in which they invest time and energy (Vallerand, 2008). This passion towards an activity is partially derived from the self-determination theory, which states that for psychological growth, people need to satiate the basic psychological requirements of autonomy, competence, and relatedness (Deci and Ryan, 1985). Thus, when people interact with the environment and engage in activities, it is to meet those needs and develop a sense of identity. As a consequence of this interaction, the elements of that environment are internalized, and the self becomes composite over time (Qadeer et al., 2016). In this line of thought, a dualistic approach to passion is proposed, that is, it can be divided between two types, harmonious and obsessive passion, which are distinguished in terms of how the activity is internalized into the individual’s own identity (Vallerand et al., 2003).

Harmonious passion results from an autonomous internalization of the activity into the individual’s identity (Vallerand et al., 2003), as individuals who feel this type of passion for their work activities experience a greater sense of cohesion between what they do and who they are, consequently leading to greater prospection in their workplace (Forest et al., 2011). Harmonious passion is associated with positive organizational outcomes such as high levels of well-being (Forest et al., 2012), work engagement, task performance, flourishing (Vallerand et al., 2003; Forest et al., 2011; Ho et al., 2011), performance (Forest et al., 2011; Ho et al., 2011), job satisfaction (Houlfort et al., 2013), work engagement (Birkeland and Buch, 2015), and job crafting (Wrzesniewski and Dutton, 2001). It relates negatively to turnover intention and burnout (Vallerand, 2010; Birkeland and Buch, 2015; Gong et al., 2020).

Obsessive passion results from a controlled internalization of the activity into the person’s identity (Vallerand et al., 2003; Vergauwe et al., 2022). It originates from intra- or interpersonal pressure due to certain contingencies linked to the activity and feelings of social acceptance or self-esteem or because the sense of arousal derived from involvement with the activity becomes uncontrollable (Vallerand et al., 2003). Obsessiveness can provide negative consequences, including psychological malaise and rumination (Forest et al., 2011; Vallerand et al., 2014), turnover intention (Gong et al., 2020), and burnout (Carbonneau et al., 2008; Birkeland and Buch, 2015; Gong et al., 2020).

Given the work passion model, there are conceptual differences between the model and work engagement (Birkeland and Buch, 2015). First, when an individual experiences passion for work, it becomes part of their self-concept (Donahue et al., 2009) and is not a prerequisite for the experience of work engagement. These two concepts describe two separate processes. On the one hand, passion for work describes the relationship with work that defines how employees identify and think about work, and on the other, engagement primarily describes the experiences that employees have at work (Birkeland and Buch, 2015). Engagement can be seen as one of the indicators of a healthy life in relation to work (Araújo and Esteves, 2016).

The term engagement was first used in 1990; it can be described as people who “use various degrees of themselves, physically, cognitively, and emotionally in performing roles at work” (Kahn, 1990, p. 694). It is a mental state characterized by three key elements: vigor, which corresponds to a behavioral component with high levels of energy and persistence at work in the face of adversity; dedication, a more emotional component, which refers to being deeply involved in work and experiencing a strong sense of meaning, enthusiasm, and challenge; and absorption, a cognitive component that indicates a high level of concentration and immersion in work (Bakker and Demerouti, 2008; Pocinho and Perestrelo, 2011; Silva et al., 2015; Araújo and Esteves, 2016). Employees who experience engagement have a more compelling sense of connection with work activities; they work more consistently (vigor), engage with a sense of meaning, enthusiasm, inspiration, pride, and challenge (dedication), and feel happy in their workplace (absorption) (Kahn, 1990; Schaufeli and Bakker, 2010).

Engagement will be measured as an organizational consequence of both types of work passion since work passion is a work attitude related to the physical, emotional, and cognitive aspects of work engagement (Kahn, 1990). Its presence in an organization broadens individuals’ perceptions and cognitions of their surroundings, allowing workers to identify the resources around them more effectively and be better equipped and more strongly motivated to successfully undertake job crafting efforts (Halbesleben, 2011; De Beer et al., 2016).

The concept of job crafting was first conceived by Wrzesniewski and Dutton (2001), who defined it as altering the boundaries and conditions of tasks, relationships, and the meaning of work at the physical and cognitive levels. It is a shifting behavior that workers employ to align their jobs with their preferences, motives, and passions in order to cultivate beneficial work experiences and improve their well-being. Thus, this concept appears in place of the “one-size-fits-all” viewpoint of conventional “job design theory,” promoting, as addressed by Elshaer et al. (2023), workers’ proactive strategies to redesign their job themselves. For most workers, job crafting leads to opportunities for improved workplace life and the possibility of meaningful contributions to this environment (Grant and Parker, 2009). In this sense, it will also be measured as consequential because passion for work, which is an individual characteristic, influences and allows employees to assess the demands and resources of their work (Vallerand and Houlfort, 2003; Vallerand, 2008), leading them to evaluate the type of investment and application of job crafting in a work context. The Job Demands–Resources (JD-R) model explains the relationship between the work demands and resources that impact employees’ well-being and job performance (Demerouti et al., 2001; Tims et al., 2012). Demands refer to a job’s physical, psychological, social, or organizational aspects that require effort and may have psychological and physiological costs for the individual. Resources, in contrast, are the physical, psychological, social, and organizational aspects that improve achievements and reduce job demands and associated costs.

Based on the JD-R model, Tims et al. (2012) propose that job crafting consists of four dimensions: (1) increased structural job resources (e.g., autonomy and variety), (2) increased social job resources (e.g., social support and feedback), (3) increased challenging job demands (e.g., new projects), and (4) decreased job demands (e.g., fewer cognitive demands). According to Berg et al. (2010), the methods workers use to achieve work passion through job crafting include investing their attention, time, and energy in working on tasks of personal interest, taking on extra tasks, or reshaping their personal work goals. In turn, passion for work, being an individual characteristic, will influence and enable employees to make assessments about the demands and resources of their work (Vallerand et al., 2003; Vallerand, 2008).

According to the JD-R model, job crafting can alter the level of demands of the job (Chia and Chu, 2017), so job crafters can either increase the level of challenging demands of the job, with new tasks, or decrease them, e.g., less cognitive demands, or both (Yen et al., 2018). Given the stress experienced due to work demands, highly invested individuals may become engrossed and lack the competence to step away from these demands, which consequently may cause feelings of additional pressure and responsibility, culminating in an excessive sense of obligation and giving rise to an obsessive passion for work (Teng, 2019). In the study by Bakker et al. (2014), it was found that workers’ initiatives to alter their job resources (crafted job resources) generated a subsequent increase in structural and social job resources, and this factor was positively related to work engagement. Job crafting also relates positively to job performance because employees make changes to their jobs to enable better performance and be more efficient to achieve goals that they value or that lead to rewarding outcomes (Warr and Inceoglu, 2012).

Job performance is a central factor in organizational psychology (Austin and Villanova, 1992) as it has been a vital issue for organizations (Qadeer et al., 2016). Thus, individual performance can be defined as actions, behaviors, and outcomes through which employees contribute to organizational goals (Viswesvaran and Ones, 2000).

Lavigne et al. (2014) have stated that employees who experience harmonious passion experience more positive attitudes in their workplace, with higher perceived control and lower perceived job demands and thus a favorable relationship with job crafting. Obsessive passion, however, leads employees to perceive the characteristics of their work as threatening because they perform their work to meet internal pressures arising from controlled internalization. Hence, resources may be perceived as obstacles and demands as additional burdens (Lavigne et al., 2014). Lastly, employee job performance is a crucial factor in determining an organization’s performance (Edwards, 1991).

Given this scenario, we propose to test a theoretical model that poses work passion as a predictor of job crafting, which mediates its relationship with engagement, ultimately impacting individual performance at work. This model suggests that work passion (a positive emotional state related to one’s work) can influence the crafting of job demands and job resources, which in turn affect employee engagement (the positive psychological state that results from fulfilling psychological needs and the satisfaction of job demands and resources). The model further suggests that engagement can positively impact individual performance at work. Therefore, the following hypothesis will be considered.

### 2.1. Work passion outcomes: job crafting; engagement and individual performance at work

A harmonious passion for work is generally associated with positive outcomes (Vallerand, 2010). With this, harmonious passion is characterized by autonomous involvement in a self-defined and highly valued activity an individual enjoys (Vallerand, 2008, 2010). When employees engage in their work and derive satisfaction, they perceive that their work environment facilitates engagement with activities they enjoy. Therefore, given the passion for work model, engagement is theoretically related to harmonious passion (Birkeland and Buch, 2015).

Employees who develop obsessive passion experience more negative emotions during and after performing the activity and frustration when they are prevented from engaging in the same activity. As such, obsessive passion leads to a more conflictual form of task engagement because it comes from a more controlled internalization (Vallerand et al., 2003).

Thus, a group of H1 was formulated: Work passion affects job crafting, considering the different dimensions—H1a: Obsessive work passion affects the increase of structural job resources; H1b: Obsessive work passion affects the increase of labor job resources; H1c: Obsessive work passion affects the increase of challenging job demands; H1d: Obsessive work passion affects the decrease of harmful work demands; H1e: Harmonious work passion affects the increase of structural job resources; H1f: Harmonious work passion affects the increase of labor job resources; H1g: Harmonious work passion affects the increase of challenging job demands; H1h: Harmonious work passion affects the decrease of harmful work demands.

Harmonious passion is positively related to job performance (Ho et al., 2011; Liu et al., 2011; Ho and Pollack, 2014); it is expected to have a positive relationship with individual performance because employees persist in performing tasks and their jobs efficiently as long as their work is enjoyable, and as a result, it does not interfere with other activities in their lives (Vallerand and Houlfort, 2003). Obsessive passion relates negatively to work performance (Ho et al., 2011; Liu et al., 2011; Ho and Pollack, 2014). Employees who experience this type of passion seem to adopt performance-avoidance goals that are less conducive to good job performance (Vallerand et al., 2007; Bonneville-Roussy et al., 2011). Other studies have showed that employees with harmonious passion have an autonomous internalization of work, which leads them to choose to engage in work, and employees with obsessive passion have a controlled internalization of work, which creates internal pressure to engage in work (Zhang et al., 2022).

As for the mediating role of job crafting, job crafting refers to the action employees take to reshape their own work to better suit their skills, interests, and passions. This could include changing their tasks, adjusting their work relationships, or changing their perceptions of their work. Job crafting can act as a mediator in the relationship between passion for work and engagement at work (Teng, 2019). When individuals are passionate about their work, they are more likely to become involved in job crafting to make their work more meaningful and rewarding. This, in turn, can increase their engagement at work. In other words, passionate workers can not only be more engaged but also actively shape their jobs in ways that further increase their engagement (Sundaray, 2011). Several empirical studies support these relationships. For example, a study by Lee et al. (2016) found that individuals who crafted their jobs experienced greater job engagement, job satisfaction, and resilience. Furthermore, a study by Vallerand et al. (2014) and Wan et al. (2022) found that harmonious passion (a type of passion in which individuals freely engage in their work because they love it) predicted job elaboration, which in turn predicted job engagement. In summary, passion for work can lead to greater engagement at work, especially when individuals are able to tailor their jobs to their interests and abilities.

Considering the previous literature, we defined the H2: Passion for work affects work engagement—H2a: Obsessive passion affects vigor mediated by job crafting; H2b: Obsessive passion affects dedication mediated by job crafting; H2c: Obsessive passion affects absorption mediated by job crafting; H2d: Harmonious passion affects vigor mediated by job crafting; H2e: Harmonious passion affects dedication mediated by job crafting; H2f: Harmonious passion affects absorption mediated by job crafting.

Passion for work can increase work engagement. Employees who are passionate about their work are likely to be more engaged and to invest more of themselves in their roles and tasks. Engaged employees are typically more focused, energetic, and dedicated to their work. As such, there is a strong relation between job engagement and job performance. Engaged employees are more likely to perform better because they are invested in their work. They feel a sense of energy and enthusiasm about their tasks, are more motivated, and tend to have better problem-solving skills. This heightened level of engagement can lead to greater productivity, higher quality of work, and greater job satisfaction, all of which contribute to improved job performance (Markos and Sridevi, 2010).

As for the mediating role of work engagement, it is plausible that work engagement mediates the relationship between job passion and job performance. This is based on the reasoning that when employees are passionate about their work, they become more engaged. This higher level of engagement can lead to better job performance. In other words, passion for work can lead to greater engagement at work, which in turn can increase job performance (Chandani et al., 2016).

Studies by Bakker et al. found that job engagement positively predicts job performance, highlighting the role of job engagement in transforming job resources (such as passion for work) into better job performance (Bakker and Bal, 2010; Bakker et al., 2023). However, it is crucial to note that the exact nature of these relationships can vary based on a number of factors, including specific contexts, individual differences, and job characteristics.

Considering the literature, the H3 group was defined: Passion for Work affects individual job performance mediated by work engagement—H3a: Obsessive passion affects individual job performance mediated by work engagement; H3b: Harmonious passion affects individual job performance mediated by work engagement.

### 2.2. Engagement relations

Studies have shown that engaged workers tend to have better individual performance because they are more motivated, committed, and dedicated to their work. Furthermore, engagement can increase creativity, innovation, and collaboration, which are key factors in improving performance (Bedarkar and Pandita, 2014; Bin and Shmailan, 2015; Teo et al., 2020).

Job crafting may be an antecedent of engagement because its purpose is to positively modify tasks, relationships, and perceptions; as a result, employees may improve their satisfaction, well-being, and fulfillment, thus increasing engagement. Employees who actively pursue job crafting show more confidence and engagement at work and perform their activities better than others (De Beer et al., 2016; Van Wingerden et al., 2017). The proactive behavior of individuals causes them to increase work resources, consequently reducing demands and increasing challenges, leading them to work engagement (Nguyen et al., 2019).

Considering those previous studies, the H4 group was developed: Job crafting affects engagement—H4a: The increase of structural job resources affects vigor; H4b: The increase of structural job resources affects dedication; H4c: The increase of structural job resources affects absorption; H4d: The increase of labor job resources affects vigor; H4e: The increase of labor job resources affects dedication; H4f: The increase of labor job resources affects absorption; H4g: The increase of challenging job demands affects vigor; H4h: The increase of challenging job demands affects dedication; H4i: The increase of challenging job demands affects absorption; H4j: The decrease of harmful work demands affects vigor; H4k: The decrease of harmful work demands affects dedication; H4l: The decrease of harmful work demands affects absorption.

Individuals with high levels of engagement have higher levels of identification with their work, personal growth and development, and performance (Kahn, 1990). The H5 group was defined: Work engagement affect individual job performance—H5a: Vigor affects individual job performance; H5b: Dedication affects individual job performance; H5c: Absorption affects individual job performance.

**Figure 1:**
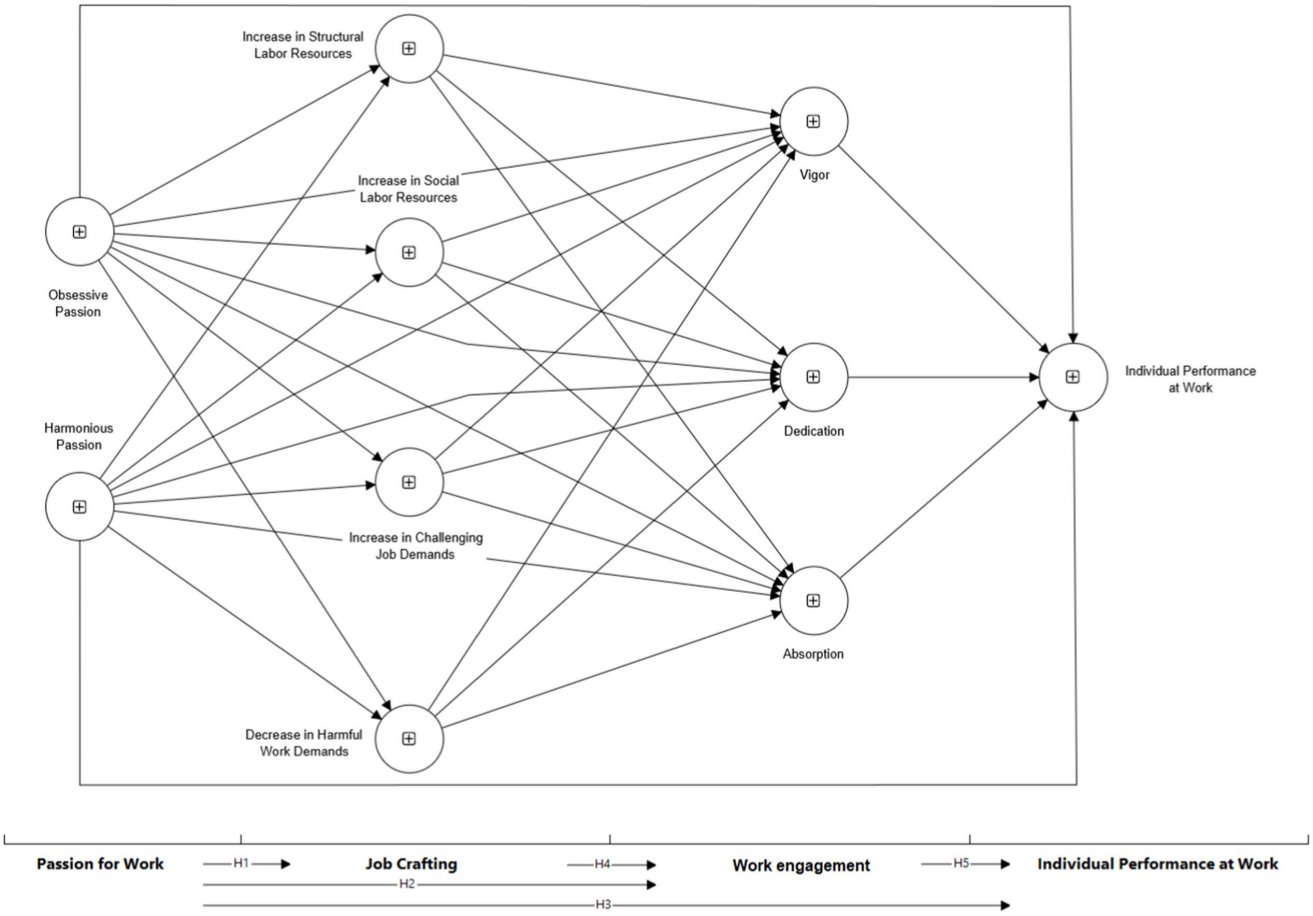
Initial structural equation model.

Figure 1 presents the proposed structural equation model.

## 3. Materials and methods

### 3.1. Design, place of study, and ethical aspects

This study employed a web survey using a non-probability convenience sample. The sample comprised 305 individuals from different branches of activity from the main regions of Portugal: 169 (55.4%) from Alentejo and Algarve, 47 (15.4%) from the Lisbon Metropolitan Area, 26 (8.5%) from the regions of Beiras, Estremadura, and Ribatejo, 26 (8.5%) from the Porto Metropolitan Area, 8 (2.6%) from Minho, Douro, and Trás-os-Montes, and 2 (0.7) from the islands of Madeira and the Azores; the remaining 27 (8.9%) did not provide this information. Data were collected through self-reported questionnaires applied digitally (Google Forms) from March to October 2020. Before filling out the questionnaire, the participants were informed of the ethical principles of anonymity and confidentiality of the data to be collected; hence, their participation was voluntary (i.e., no monetary compensation or other rewards).

### 3.2. Variables and instruments

The questionnaire was organized into two main sections: The first considered sociodemographic and socioprofessional information, and the second integrated the following self-reported measures:

The Work Passion Questionnaire (Vallerand and Houlfort, 2003), adapted to the Portuguese population by Gonçalves et al. (2014), is a 14-item scale divided into two subscales, obsessive passion and harmonious passion, and the answers were given on a 7-point Likert scale ranging from 1 (strongly disagree) to 7 (strongly agree). Harmonious passion showed high internal consistency (*α* = 0.926), and obsessive passion showed a high Cronbach’s alpha (*α* = 0.929).

The Job Crafting Scale developed by Tims et al. (2012) is composed of 21 items distributed into four dimensions: (a) increasing structural job resources (5 items), with an internal consistency of 0.902; (b) increasing social job resources (5 items), with an internal consistency of 0.906; (c) increasing challenging job demands (5 items), with an internal consistency of 0.724; and (d) decreasing hindering job demands (6 items), with an internal consistency of 0.878. The 21 items were organized on a 4-point Likert scale ranging from 0 (never) to 4 (often).

The Utrecht Work Engagement Scale aims to assess the extent to which participants are engaged with their work (Schaufeli and Bakker, 2004). In this study, the reduced version was used with nine items divided into three dimensions and three items relatable to each other: vigor, dedication, and absorption. A Likert scale with seven responses was used and composed of values from 0 (if they never had this feeling or belief) to 6 (if they felt or had it frequently). In the present sample, vigor had a Cronbach’s alpha of 0.930, dedication of 0.840, and absorption of 0.887.

The Scale of Perceived Individual Performance at Work was developed by Rego and Pina e Cunha (2008); the Portuguese version used by Rego (2009) consists of four items that allow one to assess the participants’ perception of efficiency and productivity. The answers are given on a 6-point Likert scale ranging from 0 (“the statement does not apply to me”) to 6 (“the statement completely applies to me”). The Cronbach’s alpha for this sample was 0.889.

### 3.3. Data analysis

The sociodemographic characteristics are presented as percentages, means, and standard deviations (sd). For the dimensions of the scales, Equation 1 was used so that the dimensions of the scales were comparable (Lopes, 2018):

(1) $$Ss_{i}=100*\frac{\left(Sum-Minimum\right)}{\left(Maximum-Minimum\right)}$$

where:

Ss_i_ = the standardized score for dimension i;

Sum = the sum of valid scores for dimension i;

Minimum = the lowest possible score for dimension i;

Maximum = the highest possible score for dimension i.

The scores proposed by the seminal authors of the scale were adapted to a standardized score (Ss_i_), as shown in Table 1.

**Table 1:** Adaptation of scores originally proposed by the authors of these scales with the standardized score.

Score of the original instrument	Proposed score (Ss_i_)	Classification
All dimensions	00.00–33.33	Low
	33.34–66.67	Moderate
	66.68–100.00	High

A variance-based partial least squares structural equation model (PLS-SEM) was developed (Figure 1) following the following steps: (a) structural model analysis; (b) measurement model analysis; (c) path model estimation and measurement model evaluation; (d) mediating variables analysis; and (e) structural model (Hair et al., 2017). The analyses were performed on the SmartPLS® software (version 4.0.8.5) (Ringle et al., 2022).

The measurement criteria used were internal consistency analysis, convergent validity, and discriminant validity. To fulfill the assumptions, we assumed an average variance extracted (AVE) of AVE > 0.5 and Cronbach’s alpha and composite reliability of 0.7 < θ < 0.95. Discriminant validity was assessed by the Fornell–Larcker and heterotrait–monotrait ratio (HTMT) criteria using the bootstrapping technique with 5,000 subsamples. For the Fornell–Larcker criterion, the $\sqrt{AVE}$ should be greater than the correlation matrix values and HTMT criterion, and the upper bounds of the estimated HTMT values should be below 1.0 (Hair et al., 2017).

## 4. Results

### 4.1. Sociodemographic information

The sample consisted of 305 participants aged between 19 and 63 years ( $\bar{x}\;$ = 36.06, sd = 10.41), being 54.1% (*n* = 165) female and 45.9% (*n* = 140) male. As for their marital status, 40.0% (*n* = 122) of the participants reported being single, 35.4% (*n* = 108) were married, 19.0% (*n* = 58) were in a common-law marriage, 4.9% (*n* = 15) were divorced, and the remaining 0.7% (*n* = 2) were widowed. Of the participants surveyed, 94.1% (*n* = 287) were Portuguese, and 5.9% (*n* = 18) were from other European countries.

Data on the participants’ level of education showed that 43.9% (*n* = 134) had a university degree, 28.5% (*n* = 8) completed only their high school education, 13.8% (*n* = 42) had a master’s degree, 6.6% (*n* = 20) had a specialization, 4.3% (*n* = 13) only had an elementary school education, 2.6% (*n* = 8) had a PhD, and 0.3% (*n* = 1) only had an early childhood education. As for professional activity, the participants were categorized according to the professional groups of the 2010 Portuguese classification of occupations prepared by the National Institute of Statistics (NIS). Hence, 47.2% (*n* = 144) of the individuals were specialists in intellectual and scientific activities, 14.1% (*n* = 43) were personal service, protection, and sales workers, 13.4% (*n* = 41) were technicians and intermediate-level workers, 9.8% (*n* = 30) were representatives of the legislative branch and executive bodies, directors, and executive managers, 6.9% (*n* = 21) held administrative positions, 3.3% (*n* = 10) were skilled workers from industry, construction, and craftsmen, 2.3% (*n* = 7) were from the armed forces, and 1.6% (*n* = 5) were machine/plant operators and assembly workers; unskilled workers accounted for 1.3% (*n* = 4). Of the 305 respondents, 78.0% (*n* = 238) did not hold managerial positions, while 22.0% (*n* = 67) held managerial positions.

For the time employed in their workplace, the results were quite heterogeneous, ranging from 1 to 45 years of service ( $\bar{x}\;$ = 6.69, sd = 7.02). Regarding the characterization of the organization in which the respondents worked, 44.9% (*n* = 137) worked in a national private company, 24.9% (*n* = 76) in a public company, 13.1% (*n* = 40) in a multinational company headquartered in Portugal, and 4.9% (*n* = 15) in a multinational company headquartered outside the country, while 4.6% (*n* = 14) were civil servants, 4.3% (*n* = 13) worked in the third sector, and 3.0% (*n* = 9) in local public administration.

### 4.2. Model fit tests

The model stabilized after five interactions. This study adopted several criteria to evaluate the fit of the PLS-SEM, including standardized root mean square residuals (SRMR), squared Euclidean distance (d_ULS_), geodesic distance (d_G_), and normed fit index (NFI). The results confirmed that the suggested structural model fit the data well with acceptable indices such as SRMR = 0.076, *d*_ULS_ = 8.667, *d*_G_ = 2.255, and NFI = 0.842. The SRMR value was below the threshold of 0.08, and the NFI value was above the suggested value of 0.8 (Henseler et al., 2016), indicating that the structural model satisfactorily fit the requirement.

### 4.3. Internal consistency reliability

The internal consistency among the indicators of each dimension was verified using Cronbach’s alpha value (*α*) and composed reliability (*ρ*_c_). Table 2 shows α values ranging from 0.724 to 0.930 and *ρ*_c_ between 0.817 and 0.945, higher than 0.7 and lower than 0.95 (Hair et al., 2017). Lastly, the AVEs ranged from 0.528 to 0.877, and according to the authors, values above 0.5 are suggested. These indicators ensured reliability in the internal consistency of the model dimensions.

**Table 2:** Model evaluation.

Dimension/Indicators	Factor loading	α	ρ_c_	AVE
Obsessive passion (OP)		0.929	0.943	0.702
OP_01	0.789			
OP_02	0.847			
OP_03	0.782			
OP_04	0.870			
OP_05	0.832			
OP_06	0.845			
OP_07	0.897			
Harmonious passion (HP)		0.926	0.938	0.686
Hp_01	0.759			
Hp_02	0.860			
Hp_03	0.760			
Hp_04	0.821			
Hp_05	0.922			
Hp_06	0.931			
Hp_07	0.721			
Increase in structural labor resources (IStLR)		0.902	0.930	0.731
Istlr_01	0.937			
Istlr_02	0.904			
Istlr_03	0.901			
Istlr_04	0.884			
Istlr_05	0.606			
Increase in social labor resources (ISoLR)		0.906	0.930	0.727
Isolr_01	0.829			
Isolr_02	0.858			
Isolr_03	0.896			
Isolr_04	0.863			
Isolr_05	0.816			
Increase challenging job demands (ICJD)		0.724	0.817	0.528
Icjd_02	0.666			
Icjd_03	0.750			
Icjd_04	0.741			
Icjd_05	0.747			
Decrease in harmful work demands (DHWD)		0.878	0.930	0.817
Dhwd_01	0.635			
Dhwd_02	0.908			
Dhwd_03	0.862			
Dhwd_04	0.821			
Dhwd_05	0.837			
Dhwd_06	0.762			
Vigor (VIG)		0.930	0.945	0.877
Vig_01	0.942			
Vig_02	0.946			
Vig_03	0.921			
Dedication (DED)				
Ded_01	0.890	0.840	0.904	0.758
Ded_02	0.851			
Ded_03	0.871			
Absorption (ABS)		0.887	0.930	0.817
Abs_01	0.894			
Abs_02	0.945			
Abs_03	0.870			
Individual performance at work (IPW)		0.889	0.923	0.702
Ipw_01	0.894			
Ipw_02	0.842			
Ipw_03	0.850			
Ipw_04	0.878			

### 4.4. Discriminant validity

The Fornell–Larcker and HTMT ratio criteria assessed discriminant validity to evaluate whether the measure of one dimension differs from the other (Table 3; Fornell and Larcker, 1981). We observed that the smallest root of the AVE (0.727) was higher than the highest Pearson’s correlation (OP × IPV, *r* = 0.696). The HTMT criterion, for its upper bounds, had values below 1.0 (95% confidence), so the evaluation of discriminant validity between the dimensions met the requirements. Finally, the evaluations of the measurement model for internal consistency reliability, convergent validity (Table 2), and discriminant validity (Table 3) met their requirements, empirically validating the suitability of the measurement model for the proposed model.

**Table 3:** Fornell–Larcker and HTMT criterion of the factor model.

Dim.	$\sqrt{AVE}$	Pearson’s correlation matrix									
		ABS	DHWD	DED	HP	ICJD	ISoLR	IStLR	IPW	OP	VIG
ABS	0.904	1.000									
DHWD	0.797	−0.662	1.000								
DED	0.871	0.610	−0.745	1.000							
HP	0.828	0.288	−0.512	0.234	1.000						
ICJD	0.727	0.446	−0.434	0.451	0.108	1.000					
ISoLR	0.853	0.503	−0.571	0.432	0.591	0.300	1.000				
IStLR	0.855	0.645	−0.630	0.629	0.492	0.305	0.461	1.000			
IPW	0.866	0.681	−0.658	0.652	0.461	0.348	0.506	0.625	1.000		
OP	0.838	−0.622	0.676	−0.648	−0.349	−0.381	−0.487	−0.665	−0.696	1.000	
VIG	0.936	0.692	−0.662	0.656	0.349	0.367	0.456	0.657	0.685	−0.683	1.000
UL*(HTMT)_97.5%_		
DHWD		0.930									
DED		0.973	0.924								
HP		0.408	0.639	0.358							
ICJD		0.605	0.600	0.648	0.276						
ISoLR		0.669	0.738	0.610	0.688	0.484					
IStLR		0.894	0.967	0.893	0.602	0.445	0.610				
IPW		0.846	0.913	0.822	0.574	0.510	0.677	0.882			
OP		0.960	0.916	0.991	0.451	0.526	0.632	0.892	0.829		
VIG		0.922	0.906	1.002	0.450	0.510	0.607	0.882	0.826	0.974	

*UL, upper limit.

### 4.5. Multicollinearity and predictive capability evaluation

The variance inflation factor is a statistical measure used to assess the degree of collinearity between dimensions in multiple regression models. It is used to detect multicollinearity, which is a problem that occurs when dimensions are highly correlated with each other (Thompson et al., 2017; Shrestha, 2020). A high correlation between dimensions can lead to unstable and unreliable structural coefficients (βs) (Hancock and Mueller, 2001).

The model’s predictive ability was tested by *R*^2^ and *Q*^2^, and the coefficient of explanation (*R*^2^) comes to be the proportion of variance absorbed by the endogenous dimension from the exogenous dimensions (Hair et al., 2017). As explained by the authors, *R*^2^ values vary from 0 to 1, and a value of *R*^2^ > 0.19 indicates a higher level of predictive accuracy for the dimension (strong effect), and 0.075 to 0.19 indicates a moderate effect. However, the *Q*^2^ analyzed by the blindfolding technique is a measure of the model’s quality in predicting future values based on the information provided by the exogenous dimensions (Hair et al., 2020). Based on the suggestion of Hair et al. (2017) and Lopes et al. (2020), if the value of *Q*^2^ > 0.075, the more reliable the predictions made by the model will be.

Table 4 shows that the variance inflation factor values were below 5, indicating a possible non-collinearity among the dimensions. The *R*^2^ values show an excellent degree of prediction, ranging from 0.146 to 0.799, indicating that the model’s predictive accuracy is moderate to strong (Henseler et al., 2009; Lopes et al., 2020). The statistical results showed that all *Q*^2^ values produced for each dimension were significant (i.e., *Q*^2^ > 0.075), implying the excellent predictive relevance of the model proposed in this study.

**Table 4:** Evaluation of predictive accuracy and predictive relevance.

Exogenous dimension	Endogenous dimension							
	ABS	DHDW	DED	ICJB	ISoLR	IStLR	IPW	VIG
ABS							3.630	
DHWD	3.630		3.630					3.630
DED							3.984	
HP	1.827	1.138	1.827	1.138	1.138	1.138	1.190	1.827
ICJD	1.304		1.304					1.304
ISoLR	1.918		1.918					1.918
IStLR	3.909		3.909					3.909
IPW								
OP	3.036	1.138	3.036	1.138	1.138	1.138	3.019	3.036
VIG							3.771	
*R*^2^ (value of *p*)	0.753 (0.000)	0.669 (0.000)	0.774 (0.000)	0.146 (0.000)	0.439 (0.000)	0.642 (0.000)	0.585 (0.000)	0.799 (0.000)
*Q*^2^	0.670	0.659	0.719	0.127	0.430	0.634	0.527	0.729

**Figure 2:**
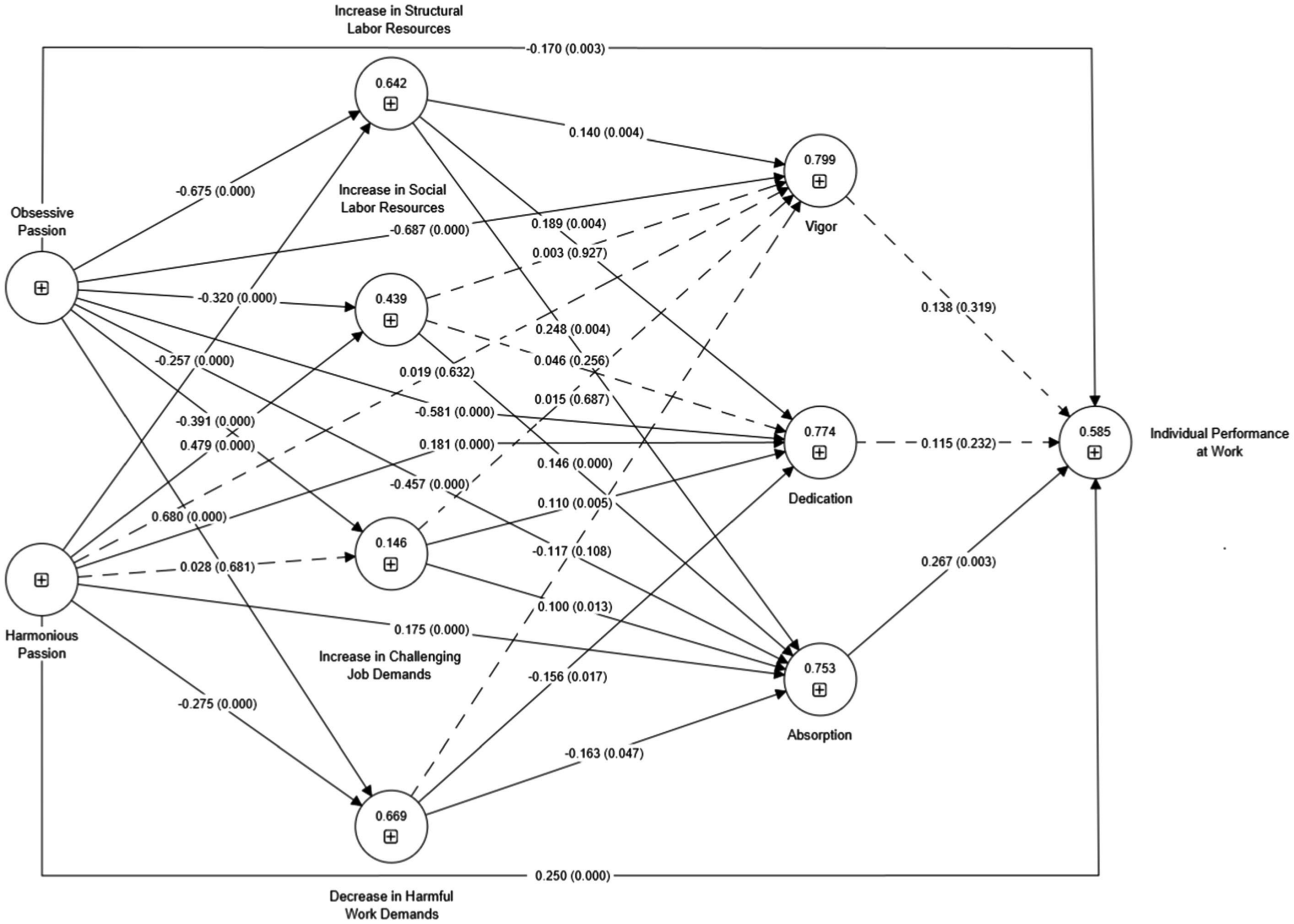
Final structural equation model.

The direct relationships among the dimensions (Table 5) and indirect effects (Table 6) were evaluated through the significance of the structural coefficients (β’s or such), and the bootstrapping technique was used (5,000 subsamples) to evaluate the significance of the coefficient values based on the value of the *t*-test. For Hair et al. (2017), the path relationship is significant for 5% significance levels when the *t*-test value exceeds 1.96. As for hypothesis H1, only the harmonious passion hypothesis with increasingly challenging work demands was rejected (*p* > 0.05). For hypothesis H2, the relationship between harmonious passion and vigor was rejected (*p* > 0.05); however, evaluating the indirect effects, it was observed that the ISoLR, ICDJ, and DHWD were the mediators of non-significance. In group H3, all the hypotheses were accepted, and when evaluating mediation for both OP → IPW and HP → IPW, absorption was the significant mediator (OP → ABS → IPW). In group H4, 4 of the 12 hypotheses were rejected (i.e., IsoLR → VIG, IsoLR → DED, ICJD → VIG, and DHWD → VIG) and did not show significant relationships (*p* > 0.05). Lastly, in group H5, vigor and dedication were unrelated to individual job performance (*p* > 0.05). Figure 2 summarizes the accepted and rejected relationships for the proposed model.

**Table 5:** Results of direct effects among dimensions.

Hypothesis/Path relation direct		Path coefficient	sd	*T*-statistic	Value of p	Result
H1a	OP → IStRL	−0.675	0.036	18.925	0.000	Supported
H1b	OP→ ISoLR	−0.320	0.055	5.821	0.000	Supported
H1c	OP → ICJD	−0.391	0.062	6.263	0.000	Supported
H1d	OP→ DHWD	0.680	0.040	17.113	0.000	Supported
H1e	HP → IStRL	0.257	0.048	5.319	0.000	Supported
H1f	HP → ISoLR	0.479	0.045	10.701	0.000	Supported
H1g	HP → ICJD	0.028	0.068	0.411	0.681	Not supported
H1h	HP → DHWD	−0.275	0.048	5.714	0.000	Supported
H2a	OP → VIG	−0.687	0.055	12.518	0.000	Supported
H2b	OP→ DED	−0.581	0.063	9.223	0.000	Supported
H2c	OP → ABS	−0.457	0.110	4.167	0.000	Supported
H2d	HP → VIG	0.019	0.039	0.480	0.632	Not supported
H2e	HP → DED	0.181	0.043	4.228	0.000	Supported
H2f	HP → ABS	0.175	0.049	3.588	0.000	Supported
H3a	OP → IPW	−0.170	0.072	2.361	0.003	Supported
H3b	HP → IPW	0.250	0.045	5.577	0.000	Supported
H4a	IStRL → VIG	0.140	0.049	2.875	0.004	Supported
H4b	IStRL → DED	0.189	0.065	2.896	0.004	Supported
H4c	IStRL → ABS	0.248	0.086	2.887	0.004	Supported
H4d	ISoLR → VIG	0.003	0.037	0.092	0.927	Not supported
H4e	ISoLR → DED	0.046	0.041	1.137	0.256	Not supported
H4f	ISoLR → ABS	0.146	0.041	3.531	0.000	Supported
H4g	ICJD → VIG	0.015	0.036	0.402	0.687	Not supported
H4h	ICJD → DED	0.110	0.039	2.785	0.005	Supported
H4i	ICJD → ABS	0.100	0.040	2.497	0.013	Supported
H4j	DHWD → VIG	−0.117	0.073	1.607	0.108	Not supported
H4k	DHWD → DED	−0.156	0.065	2.396	0.017	Supported
H4l	DHWD → ABS	−0.163	0.082	1.988	0.047	Supported
H5a	VIG → IPW	0.138	0.139	0.996	0.319	Not supported
H5b	DED → IPW	0.115	0.096	1.194	0.232	Not supported
H5c	ABS → IPW	0.267	0.091	2.946	0.003	Supported

sd, standard deviation.

**Table 6:** Results of indirect effects among dimensions.

Hypothesis / Path relation Indirect		Path coefficient	sd	T-statistic	*value of p*	Result
H2a01	OP → IStLR → VIG	0.095	0.034	2.772	0.006	Supported
H2a02	OP → ISoLR → VIG	−0.001	0.012	0.090	0.928	Not supported
H2a03	OP → ICJD → VIG	0.006	0.015	0.390	0.697	Not supported
H2a04	OP → DHWD → VIG	0.080	0.050	1.581	0.114	Not supported
H2b01	OP → IStLR → DED	0.168	0.057	2.923	0.003	Supported
H2b02	OP → ISoLR → DED	0.047	0.015	3.039	0.002	Supported
H2b03	OP → ICJD → DED	0.039	0.016	2.374	0.018	Supported
H2b04	OP → DHWD → DED	0.111	0.056	1.969	0.049	Supported
H2c01	OP → IStLR → ABS	0.127	0.044	2.885	0.004	Supported
H2c02	OP → ISoLR → ABS	0.015	0.013	1.108	0.268	Not supported
H2c03	OP → ICJD → ABS	0.043	0.018	2.359	0.018	Supported
H2c04	OP → DHWD → ABS	0.106	0.045	2.353	0.019	Supported
H2d01	HP → IStLR → VIG	−0.036	0.014	2.510	0.012	Supported
H2d02	HP → ISoLR → VIG	0.002	0.018	0.091	0.927	Not supported
H2d03	HP → ICJD → VIG	0.000	0.003	0.143	0.886	Not supported
H2d04	HP → DHWD → VIG	−0.032	0.021	1.523	0.128	Not supported
H2e01	HP → IStLR → DED	−0.048	0.019	2.594	0.010	Supported
H2e02	HP → ISoLR → DED	−0.022	0.020	1.088	0.276	Not supported
H2e03	HP → ICJD → DED	0.003	0.008	0.369	0.712	Not supported
H2e04	HP → DHWD → DED	−0.043	0.019	2.253	0.024	Supported
H2f01	HP → IStLR → ABS	−0.064	0.022	2.867	0.004	Supported
H2f02	HP → ISoLR → ABS	−0.070	0.022	3.221	0.001	Supported
H2f03	HP → ICJD → ABS	0.003	0.007	0.406	0.685	Not supported
H2f04	HP → DHWD → ABS	−0.045	0.022	2.045	0.032	Supported
H3a01	OP → VIG → IPW	0.095	0.094	1.014	0.310	Not supported
H3a02	OP → DED → IPW	0.067	0.056	1.181	0.237	Not supported
H3a03	OP → ABS → IPW	0.122	0.056	2.184	0.029	Supported
H3b01	HP → VIG → IPW	0.003	0.008	0.317	0.751	Not supported
H3b02	HP → DED → IPW	0.021	0.019	1.100	0.271	Not supported
H3b03	HP → ABS → IPW	0.047	0.017	2.767	0.006	Supported

sd, standard deviation.

### 4.6. Analysis of scale dimensions

**Figure 3:**
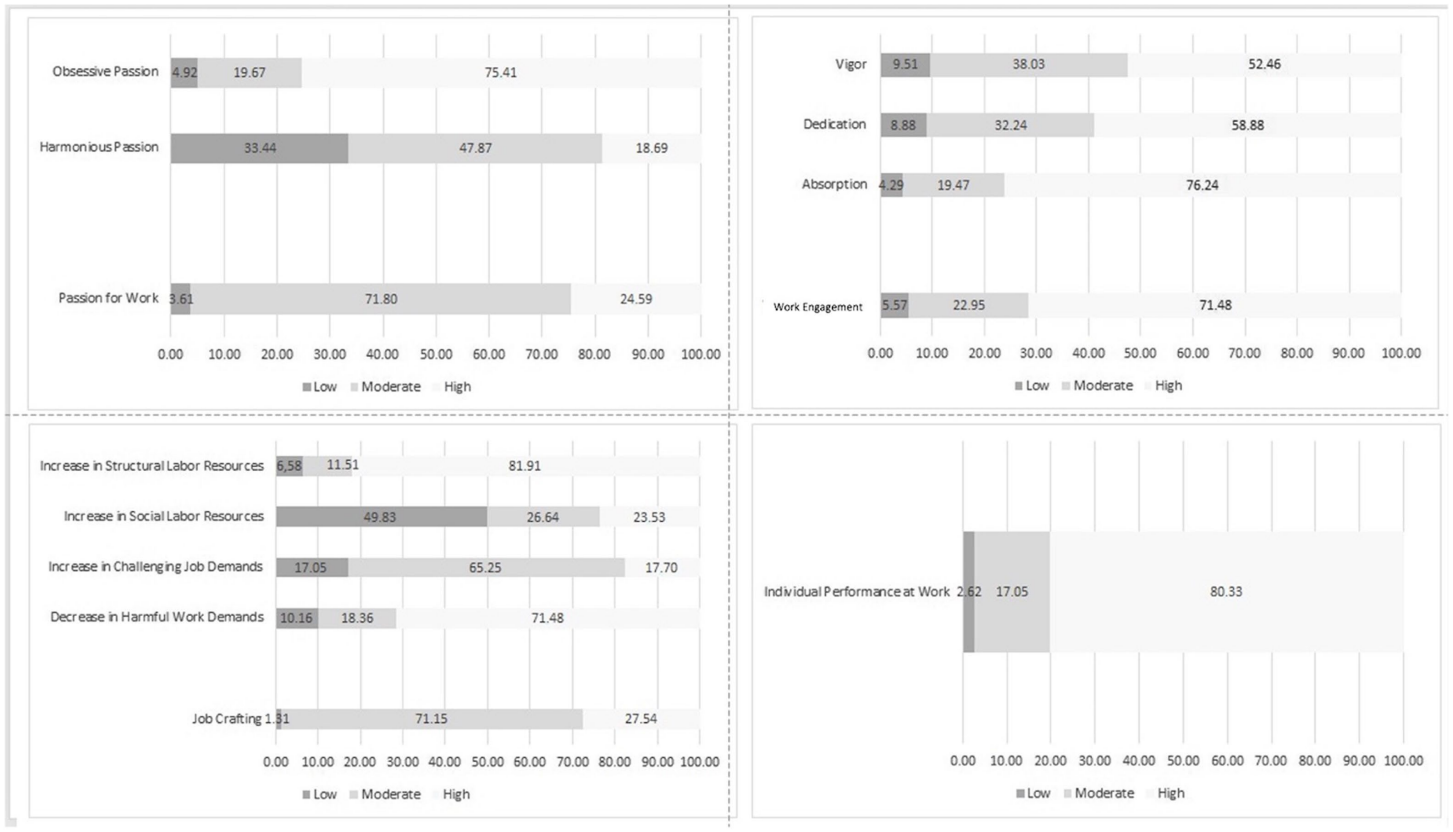
Classification of passion for work, job crafting, engagement, and individual performance.

The characteristics in relation to the dimensions of the scales are presented in Figure 3. Our findings showed a high obsessive passion of 230 (71.41%) and a moderate harmonious passion of 146 (47.87%), resulting in a moderate passion of 219 (71.80%). As for job crafting, there was a significant increase in structural job resources of 249 (81.91%) and a sharp decrease in hindering job demand of 218 (71.48%), resulting in a moderate job crafting of 217 (71.15%). Engagement, nonetheless, had high absorption of 231 (76.24%), high dedication of 179 (58.88%), and high vigor of 160 (52.46%); as a result, 245 (80.33%) of the workers exhibited high work engagement. Lastly, 245 (80.33%) of the workers had high perceived individual work performance.

## 5. Discussion and conclusion

This study analyzed the possible relationships between work passion, job crafting, work engagement, and individual job performance using PLS-SEM. This study was applied to 305 Portuguese workers from different regions of Portugal, and our findings confirmed that the model assumptions were met (i.e., the internal consistency, convergent validity, discriminant validity, and multicollinearity of the dimensions did not reach values outside tolerable limits).

By evaluating the first hypothesis (H1), we observed that obsessiveness negatively impacts job crafting, which refers to adapting to tasks and responsibilities in the work environment (Lavigne et al., 2014). Obsessive passion may lead workers to excessive fixation, impairing their abilities to view work in an expanded and flexible way and missing out on opportunities for growth and development (Patel et al., 2015; Wan et al., 2022). Obsession may induce workers to adopt impulsive and destructive behavior, damaging interpersonal relationships, especially their professional image (Bowen, 2020). As for harmonious passion, it was not significantly related to the increase in challenging work demands, which means it leads the worker to positive outcomes with high levels of engagement, motivation, and satisfaction, increasing performance and well-being (Teng, 2019; Jan and Zainal, 2020). Harmonious passion can be a positive factor in the work environment, although its relationship with challenging demands is complex and depends on multiple factors (Salas-Vallina et al., 2020).

In the second hypothesis (H2), we found that the obsessive passion dimension negatively leads to the engagement dimensions, showing that attachment-oriented activities characterize obsessiveness and may cause negative outcomes such as burnout and low job satisfaction, consequently reducing engagement (Choi et al., 2020; Tóth-Király et al., 2021). Obsessive passion may damage employees’ potential because the fixation causes them to stop doing other activities, therefore damaging their professional reputations. A balance between passion and engagement in the workplace prevents these behaviors from harming personal well-being (Bowen, 2020; Devine et al., 2020; Horwood et al., 2021; Zhu et al., 2022). As for harmonious passion not being related to vigor, this is quite possible since harmonious passion refers to an intense, controlled, and balanced feeling, while vigor refers to energy, strength, and intensity; thus, a harmonious person does not necessarily need to be at their “peak” vigor (Blanco-Donoso et al., 2020; Oktavia et al., 2020; Smith et al., 2023). As for the mediation of job crafting between passion for work and work engagement, absorption was the dimension that best interacted with the dimensions of the Job Crafting Scale, accepting three sub-hypotheses with obsessive passion and two sub-hypotheses with harmonious passion. It can be hypothesized that passion for work can increase work absorption, which in turn can lead to a higher overall level of work engagement. This would be based on the idea that passionate workers can become more deeply absorbed in their work, contributing to a broader experience of engagement (Zigarmi et al., 2009; Astakhova et al., 2022).

In the third hypothesis (H3), we observed that obsessive passion negatively relates to individual performance, which means that compulsively obsessing over something may affect individual performance. Obsession leads to distraction or time demands, impairing the ability to concentrate and accomplish tasks at work (Lucas et al., 2019; Choi et al., 2020; Pollack et al., 2020; Ho et al., 2021). The anxiety and stress resulting from obsession negatively affect the individual’s mental and physical health, ergo impairing work performance (Damirchi et al., 2020; Manning et al., 2021; Serrano-Fernandez et al., 2021; Vismara et al., 2021). As for the relationship between harmonious passion and individual performance, this may occur because harmonious passion affects the ability to concentrate and accomplish tasks (Chummar et al., 2019; Schenkel et al., 2019; Teng, 2019); therefore, passion and job responsibilities must be balanced to improve individual performance (Pollack et al., 2020). In the same way as the previous mediation, absorption is the dimension that mediates the relationship between passion for work and individual performance at work. Therefore, it is plausible that absorption may mediate the relationship between job passion and job performance. Passionate workers may be more likely to experience high levels of absorption, and this deep involvement in their tasks can lead to better job performance (Vallerand et al., 2014).

As for the fourth hypothesis (H4), the increase in social job resources is not related to vigor and dedication because social job resources involve conscious changes in interpersonal relationships at work in search of new learning opportunities and personal development, while engagement, more specifically vigor, refers to the level of energy, enthusiasm in performing activities, and dedication to achieving the goals and objectives of the organization (Sajjad and Shahbaz, 2020; Al-Hamdan and Bani Issa, 2022; Srimulyani and Hermanto, 2022; Nurtjahjani et al., 2023). In contrast, the significant relationship between social job resources and absorption is confirmed as workers with good interpersonal relationships and opportunities for learning and growth tend to feel more engaged and absorbed in their work activities (Rofcanin et al., 2019; Balakrishnan and Dwivedi, 2021).

Therefore, it was possible to empirically validate the relationships proposed in the model, remembering that other factors such as work resources, work environment, and personal characteristics can also influence these relationships.

Challenging job demands do not relate to vigor; this is very complex to state, as there may be influences from external factors (e.g., subjective perceptions) and internal factors (e.g., personality, resilience, and coping style) (Alonso-Tapia et al., 2019; Ngui and Lay, 2020; Leguizamo et al., 2021). Nevertheless, it is known that challenging demands are perceived to be excessive in relation to the available resources, so they could reduce workers’ vigor, leading to emotional exhaustion and increasing vigor through the possibility of personal growth and development (Sahi et al., 2022; Toyama et al., 2022).

As for the decrease in harmful work demands being unrelated to vigor, this is because the decrease in demands negatively and positively impacts on vigor, i.e., excessive work hours, pressure to achieve goals, and lack of support from the organization can lead to stress loads and mental exhaustion. In contrast, decreasing the burden of harmful demands may improve workers’ emotional health and well-being and increase vigor (Kinnunen et al., 2019; Riedl and Thomas, 2019).

Lastly, the final hypothesis (H5) demonstrated that vigor and dedication are not predictors of individual job performance. This can be justified by the bi-directionality of the relationships between engagement dimensions with individual performance (Wood et al., 2020; Santalla-Banderali and Alvarado, 2022; Tan et al., 2022). Nonetheless, absorption was significantly related to performance because when workers are highly absorbed, they tend to devote more time to work with positive attitudes and feel more professionally fulfilled. As a result, it significantly affects individual performance (Kuijpers et al., 2020; Jaya and Ariyanto, 2021).

By analyzing the Ssi ratings for each dimension (Figure 3), we found the Portuguese workers had a high obsessive passion for work (71.41%), which refers to a high degree of enthusiasm and dedication for the work they do, showing that these workers can feel work passion while balancing priorities and other activities in their life (Ho et al., 2011; Salas-Vallina et al., 2020). This will reflect in high individual performance (80.33%), although it is important to balance the passion for what they do with their personal life in order to avoid burnout and maintain a healthy lifestyle (Brown et al., 2021).

As for work engagement, a high absorption (76.24%) stood out, referring to the fact that workers are involved and focused on their activities, and this reflected in the high individual performance (80.33%) due to dedication and motivation (Meijerink et al., 2020). However, excessive absorption may lead to stress and burnout and, as a result, affect performance, reinforcing a balance in work involvement with time for rest and leisure (Nerstad et al., 2019).

Finally, a high increase in structural labor resources (81.91%), which refers to safe working conditions, appropriate tools, adequate training, social support, and effective supervision that will positively affect individual performance, was observed (De Brier et al., 2020). However, a high decrease in hindering job demands (71.48%) reflects on work overload regarding responsibilities, generating interpersonal conflicts (Gu et al., 2020). Therefore, an ideal scenario would be that the increase in labor resources would reduce harmful demands, remembering that in this study, both situations undergo a high absorption to have high individual job performance (Shin et al., 2020; Harju et al., 2021).

This study aimed to shed more light on the impact of work passion on individual job performance associated with job crafting and work engagement. In this sense, our findings support the premise that harmonious passion relates positively to the consequences under study—engagement, job crafting, and perceived individual performance—and that obsessive passion relates negatively to them. This study also showed that both types of work passion, engagement, and job crafting contribute to the explanation of perceived individual performance. The results emphasize the importance of positive work environments and their contribution to performance. This study is believed to contribute to a model study of developing a passion for work, as empirical evidence was found between harmonious and obsessive passion and organizational consequences. In terms of practical implications, our results may suggest the importance of companies promoting work–life balance strategies and recovery experiences, so that employees are harmoniously passionate about their work and, consequently, their attitudes towards work are positive. One way to work and promote these healthy organizational environments may be to promote strategies that encourage job crafting.

For future studies, a longitudinal study should be developed to link work passion with the organizational outcomes from this study. This is necessary because harmonious and obsessive passion are internalized in the identity of individuals, and they are not static and can change over time (Ethier and Deaux, 1994). Another aspect is that there may be other conditions that affect the relationship between the two types of passion for work and organizational consequences, which presents an exciting research opportunity for future studies to consider organizational support or constraints, the personal characteristics of respondents (e.g., self-esteem), and organizational culture supporting work–life balance (Burke and Singh, 2014). In another way, it could be interesting to add new variables; for example, as suggested by Elshaer et al. (2022a,b), future research may also focus on humor as a leadership tool to improve outcomes or consider the role of green perceived organizational support.

### 5.1. Limitations and future research

Further research is crucial to better understand the impact of work passion on organizational outcomes. It is of the utmost importance for organizations to be concerned with promoting and maintaining workers’ mental health, and the optimal functioning of the institution should promote harmonious passion, as opposed to obsessive passion, as this is associated with positive organizational outcomes since, when witnessed in the work context, it relates to workers’ mental health, vitality, and affective commitment, which are mediated by satisfying the basic needs for autonomy and competence (Forest et al., 2011).

## Data availability statement

The raw data supporting the conclusions of this article will be made available by the authors, without undue reservation.

## Ethics statement

Ethical review and approval was not required for the study on human participants in accordance with the local legislation and institutional requirements. The patients/participants provided their written informed consent to participate in this study.

## Author contributions

The design of the study, theoretical framework, methodology definition, and article review were carried out by JV, AG, and DR. Data collection was performed by DR, MM, and DS. The preliminary data analysis and interpretation were conducted by LL, MM, and DS. The discussion and applied interpretation of the data were carried out by JV and DR. All authors contributed to the article and approved the submitted version.

## Funding

This work was funded by national funds through the Fundação para a Ciência e a Tecnologia (FCT) as part of the CIP project (Ref. UID/PSI/04345/2020).

## Conflict of interest

The authors declare that the research was conducted in the absence of any commercial or financial relationships that could be construed as a potential conflict of interest.

## Publisher’s note

All claims expressed in this article are solely those of the authors and do not necessarily represent those of their affiliated organizations, or those of the publisher, the editors and the reviewers. Any product that may be evaluated in this article, or claim that may be made by its manufacturer, is not guaranteed or endorsed by the publisher.
